# A Hybrid Uniplanar Pedicle Screw System with a New Intermediate Screw for Minimally Invasive Spinal Fixation: A Finite Element Analysis

**DOI:** 10.1155/2020/5497030

**Published:** 2020-11-18

**Authors:** Jia Li, Li-Cheng Zhang, Jiantao Li, Hao Zhang, Jing-Xin Zhao, Wei Zhang

**Affiliations:** ^1^Department of Orthopaedics, The First Medical Center, Chinese PLA General Hospital, Beijing, China; ^2^National Clinical Research Center for Orthopaedics, Sports Medicine & Rehabilitation, Beijing, China; ^3^The Faculty of Orthopaedics, The Fourth Medical Centre, Chinese PLA General Hospital, Beijing, China

## Abstract

**Purpose:**

A hybrid pedicle screw system for minimally invasive spinal fixation was developed based on the uniplanar pedicle screw construct and a new intermediate screw. Its biomechanical performance was evaluated using finite element (FE) analysis.

**Methods:**

A T12-L2 FE model was established to simulate the L1 vertebral compression fracture with Magerl classification A1.2. Six fixation models were developed to simulate the posterior pedicle screw fracture fixation, which were divided into two subgroups with different construct configurations: (1) six-monoaxial/uniplanar/polyaxial pedicle screw constructs and (2) four-monoaxial/uniplanar/polyaxial pedicle screw constructs with the new intermediate screw. After model validation, flexion, extension, lateral bending, and axial rotation with 7.5 Nm moments and preloading of 500 N vertical compression were applied to the FE models to compare the biomechanical performances of the six fixation models with maximum von Mises stress, range of motion, and maximum displacement of the vertebra.

**Results:**

Under four loading scenarios, the maximum von Mises stresses were found to be at the roots of the upper or lower pedicle screws. In the cases of flexion, lateral bending, and axial rotation, the maximum von Mises stress of the uniplanar screw construct lay in between the monoaxial and polyaxial screw constructs in each subgroup. Considering lateral bending, the uniplanar screw construct enabled to lower the maximum von Mises stress than monoaxial and polyaxial pedicle screw constructs in each subgroup. Two subgroups showed comparable results of the maximum von Mises stress on the endplates, range of motion of T12-L1, and maximum displacement of T12 between the corresponding constructs with the new intermediate screw or not.

**Conclusions:**

The observations shown in this study verified that the hybrid uniplanar pedicle screw system exhibited comparable biomechanical performance as compared with other posterior short-segment constructs. The potential advantage of this new fixation system may provide researchers and clinical practitioners an alternative for minimally invasive spinal fixation with vertebral augmentation.

## 1. Introduction

Spinal fixation with the posterior pedicle screw-rod system has been considered the mainstream treatment method for degenerative and traumatic spinal diseases. A number of in vitro, finite element (FE) and clinical studies have been performed to figure out the influencing factors for the fixation stability and modification or alternative methods to improve the biomechanical or functional outcomes, which included bone mechanical properties [1], disc arthroplasty [2, 3], semirigid rod [4], and dynamic or hybrid stabilization device [5, 6].

Regarding thoracic and lumbar vertebral fractures, minimally invasive short-segment pedicle screw instrumentation is an easy procedure and gaining momentum as a routine option. However, limited exposure increases the difficulty of coupling pedicle screws with rods, which will lead to a superficial position of longitudinal rods and decreased strength of the entire construct. Benefited by a decreased force arm, the polyaxial pedicle screw (PAPS) allows easier assembling pedicle screws with rods and provides theoretically better biomechanical strength. Nevertheless, prior studies demonstrated that PAPS tended to slippage through the universal joint, and its mechanical strength was always less than the monoaxial pedicle screw (MAPS) in the sagittal plane [7, 8]. These phenomena allow a new uniplanar pedicle screw (UPPS) to be introduced.

As the head of UPPS that can only swivel freely in one specific plane while fixed in other directions, the freedom of movement of the screw's head in the axial plane of body can facilitate the pedicle screw-rod assembly, without sacrificing the stiffness of the construct in the sagittal plane, whereas only two studies performed the biomechanical investigation of UPPS for spinal fixation to date, which verified its biomechanical advantage over PAPS in the sagittal plane [7, 8]. Meanwhile, biomechanical and clinical studies have suggested that an intermediate screw can provide substantially improved stability of posterior short-segment constructs in spinal fracture fixation [9, 10]. Nevertheless, few studies incorporated an intermediate screw into the UPPS construct.

In addition to these modifications to the instrument design, for most thoracolumbar bursts or compression fractures, vertebral augmentation has been used as a means to mechanically support the fractured vertebra anteriorly. Nonetheless, most clinical trials utilized one-above and one-below 4-MAPS short-segment construct, of which some reported excellent reduction and lordosis maintenance [11, 12], while others concluded with indifferent results [13]. There was limited evidence about the six-screw short-segment construct with additional vertebroplasty at the fracture level. Liao et al.'s finite element analysis (FEA) study verified that additional vertebroplasty could provide a stiffer 6-MAPS construct and less stress in the thoracolumbar burst fracture model [10]. Moreover, the 6-MAPS short-segment construct was also performed with vertebroplasty [12] and bone grafting [14] in the open thoracolumbar fracture operation. However, during operation, the intermediate screw needed to be removed after distraction and reduction, followed by vertebroplasty. The current pedicle screw constructs do not allow vertebral augmentation without interrupting the procedure of reduction and fixation of the spinal fracture.

In the present study, a hybrid UPPS fixation system was introduced. With this short-segment construct, a new intermediate screw (NIS) was developed to facilitate vertebroplasty and reduction maneuver. Different from the traditional parallel configuration of pedicle screws, this NIS is inserted in a more outside-in direction. This configuration allows bilateral intermediate screws to achieve the center of the injured vertebra, elevate the depressed endplate, and then maintain its reduction position more efficiently. To demonstrate the biomechanical performance of this hybrid UPPS system, an FEA was performed to compare the monoaxial, uniplanar, and polyaxial screw short-segment constructs with or without the NIS.

## 2. Materials and Methods

### 2.1. Instrument Design

**Figure 1:**
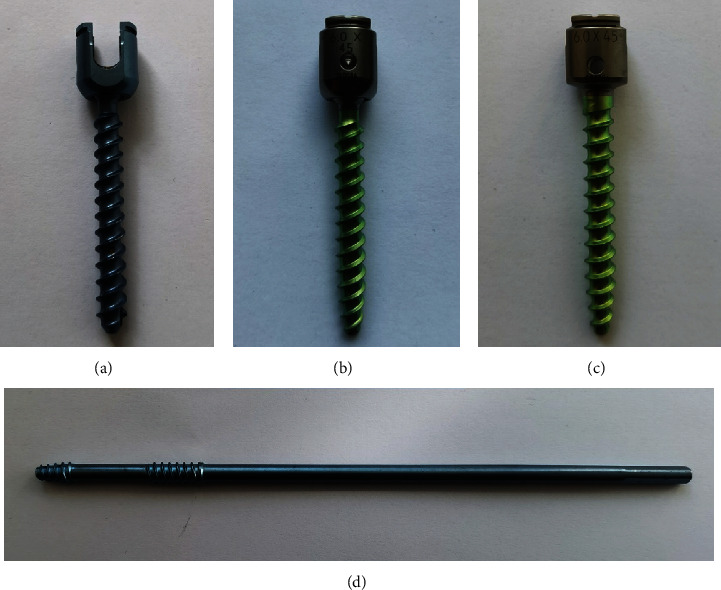
Four types of screws used in this study: (a) UPPS, (b) PAPS, (c) MAPS, and (d) NIS.

The geometric 3D model of UPPS was drawn using the Solidworks software (Dassault Systèmes, Concord, MA, USA). The UPPS is barrel designed, with the outer and inner diameter of 6 mm and 4.1 mm, respectively. The screw-tulip joint of our UPPS allows about ±30° angular motion of the screw in the axial plane of the body (perpendicular to the plane in which the slot of the gasket is located), which is greater than Ye et al.'s UPPS of ±25° [7]. During manufacture, the spot welding technique was used to minimize the mobility of the screw-tulip joint in other directions. Unlike the UPPS, the NIS was designed based on the USS® cannulated schanz screw. Our modification is that the threads of the NIS are located at the anterior and posterior one-third shank, with a smooth part in the middle. Both the UPPS and NIS have lateral fenestrations in the distal portion of the thread for bone cement or graft injection. The outer and inner diameters of the NIS are 6 mm and 5.2 mm, respectively. Besides, PAPS and MAPS (VIPER® System, DePuy Spine, Inc.) were also used in this study for comparison between four types of constructs in FEA. The thread type and pitch of the PAPS, UPPS, and MAPS are single lead and 3 mm, whereas those of the NIS are double lead and 4 mm. All four types of screws used in this study were shown in Figure 1.

### 2.2. T12-L2 FE Modeling

**Figure 2:**
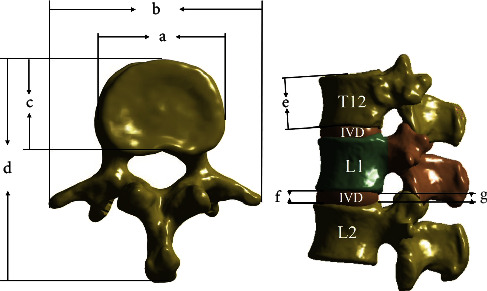
Measured anatomical parameters of vertebra and intervertebral disc (IVD): (a) transverse width of the vertebral body, (b) distance between two extreme tips of transverse processes, (c) anteroposterior width of the vertebral body, (d) distance between tip of the spinous process and the anterior wall of the vertebral body, (e) height of the vertebral body, (f) the anterior height of IVD, and (g) the posterior height of IVD.

The geometry model of T12-L2 spine was reconstructed using Mimics software (Version 20, Materialise NV, Leuven, Belgium) based on the CT-scan data in Digital Imaging and Communications in Medicine format (512 × 512 pixels/slice, slice thickness 0.625 mm) of a 32-year-old healthy male without spine pathologies. The established model in Stereo-Lithography format was inputted into Geomagic Studio software (Version 12, Research Triangle Park, Morrisville, NC, USA) for reverse engineering reconstruction. After a series of image processing, a 3D model of T12-L2 in Initial Graphics Exchange Specification format was established. The measurement method and results of dimensions of the vertebra and intervertebral disc (IVD) were shown in Figure 2 and Table 1.

The material properties of the T12-L2 FE model were assigned based on previous studies [10, 15] (Table 2). The cortical bone had a thickness of 1 mm. The posterior elements included the pedicle, spinal process, transverse process, and articular process. The interfaces between the facet joints were set as frictionless surface-to-surface contact. The interfaces between the screw and bone were set to be bonded. The contact between the head and tulip of UPPS and PAPS was set as surface-to-surface contact with a friction coefficient of 0.3. According to the attachment positions, the spinal ligaments were established and defined as nonlinear hyperplastic material using Combine 39, which only allowed for tension deformation without the compression behavior. The mechanical behaviors of the spinal ligaments were based on nonlinear stress-strain curves [16].

**Figure 3:**
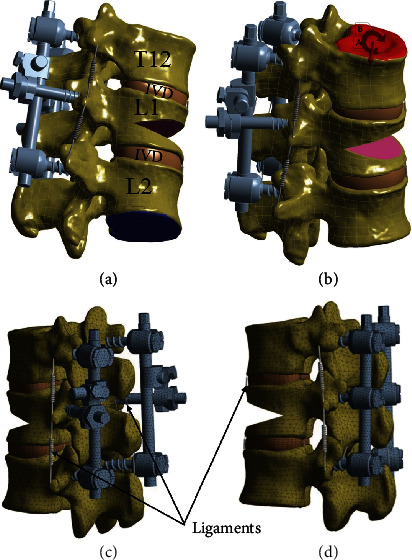
The T12-L2 finite element model of four uniplanar pedicle screw fixation with two new intermediate screws. The geometry image: (a) the inferior surface of L2 was fixed in all directions. (b) A reference point was created on the superior surface of the T12 vertebra for force and moment application. The meshing image: (c) and (d).

The bone and intervertebral disc were meshed with Solid 187 (Figure 3). Due to a variety of material properties of the spine FE model and methods of the FE modeling, a mesh sensitivity test was performed to verify the developed FE model of T12-L2. The maximum von Mises stress was used to evaluate the mesh convergence. Four T12-L2 models were generated with mesh sizes of 0.5 mm, 1 mm, 2 mm, and 3 mm for all structures, respectively. After the lower surface of L2 was fixed in all directions, a pure moment of 7.5 Nm was applied on the upper surface of T12. The maximum von Mises stresses of models with mesh size of 1 mm, 2 mm, and 3 mm were calculated and compared with that with a mesh size of 0.5 mm. When the difference was less than 5%, the mesh was considered convergent. Considering the burden and precision of calculation, the mesh size of 1 mm was chosen in further analysis. The percentage of error was 2.70% in this situation.

### 2.3. Model Validation

Before FEA was performed on the spinal fixation model, the FE model of the intact spine was validated with range of motion (ROM) of T12-L1 and L1-L2, respectively. The inferior surface of the inferior vertebra was fixed in all directions. A reference point was created on the superior surface of the T12 and L1 vertebrae for force and moment application, respectively. Flexion, extension, right lateral bending, and right axial rotation with pure 7.5 Nm moment were applied to T12-L1 and L1-L2, respectively. The ROM of two segmental units was calculated at the endpoint of loading.

### 2.4. Fixation Model

Following the previously established methods [7, 17, 18], a cuneiform osteotomy was applied to the L1 vertebra to construct the vertebral compression fracture model with Magerl classification A1.2 [19]. Six types of short-segment pedicle screw constructs were established to simulate the thoracolumbar vertebral compression fracture fixation, including three six-screw constructs with 211,632 elements and 373,160 nodes on average: (1) 6-MAPS, (2) 6-UPPS, (3) 6-PAPS, and three four-screw constructs with the NIS with 206,060 elements and 362,869 nodes on average: (1) 4-MAPS/2-NIS, (2) 4-UPPS/2-NIS, (Figure 3), and (3) 4-PAPS/2-NIS. After assembly, the fracture fixation models were imported into Ansys Workbench software (Version 18, ANSYS Inc., Pittsburgh, Pennsylvania, USA) for further Boolean operation.

### 2.5. FEA

To analyze the biomechanical properties of these six fixation models, the moments of 7.5 Nm in each direction with preloading of vertical 500 N compression were applied to the reference point of the T12 vertebra [20]. Under different types of loading, the maximum von Mises stress on the fixation construct and the endplates of T12 and L2, the ROM of T12-L2, and the maximum displacement of T12 were recorded and compared between six groups.

## 3. Results

### 3.1. FE Model Validation

The ROMs of the intact T12-L1 model were shown in Table 3. These findings were similar to those of previous studies [21, 22].

### 3.2. ROM of T12-L2 and Maximum Displacement of T12

As shown in Table 4, the ROM of T12-L2 and the maximum displacement of T12 under flexion loading were similar between the six fixation groups. Under the other three loading types, the ROM and maximum displacement were the smallest in the MAPS groups, followed by the uniplanar and polyaxial groups in sequence, irrespective of the type of the intermediate screw.

### 3.3. Von Mises Stress on the Pedicle Screw System

**Figure 4:**
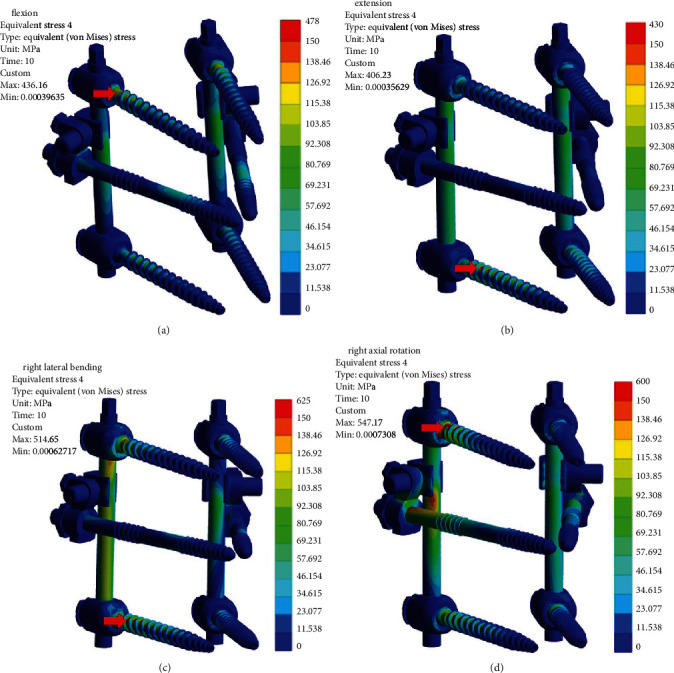
Von Mises stresses nephogram of the four-uniplanar pedicle screw construct with two new intermediate screws under different loading types. Upper left: flexion; upper right: extension; lower left: right lateral bending; lower right: right axial rotation. The maximum von Mises stress of the entire construct under loading was marked with red arrow.

Disregarding the sporadic stress concentration point caused by the screw-tulip mobile joint of UPPS, which was out of the scope of this study [23], the maximum von Mises stresses on the instrument were mainly located at the roots of the upper and lower pedicle screws under four types of loading, according to the stress nephogram of the FE model (Figure 4; Supplementary Material (available here)). Maximum von Mises stresses of three six-screw constructs and their NIS counterparts were seen at similar positions (Table 5).

Under flexion loading, the maximum von Mises stresses were seen on the upper screws of the monoaxial and uniplanar screw constructs and the lower screws of the polyaxial screw constructs. Moreover, the maximum stress was the lowest in the 6-PAPS construct, followed by the 6-UPPS and 6-MAPS construct in sequence, and the NIS subgroup displayed the same varying rule.

In the case of extension, the maximum von Mises stresses were shown on the lower screws in all six construct groups. The maximum stress was the lowest in the 6-MAPS construct, followed by the 6-UPPS and 6-PAPS construct in sequence, and the NIS subgroup displayed similar varying rule.

For the condition of lateral bending, the maximum von Mises stresses were found at the lower screws in uniplanar and polyaxial screw constructs but the upper screws in monoaxial screw constructs. Besides, the varying rule was opposite to the flexion condition.

The right axial rotation movement resulted in similar changing trends like the flexion loading, except for the pair of 4-PAPS/2-NIS and 6-PAPS. The possible reason might be that the fixed NIS altered the force-bearing condition of the polyaxial screw constructs and caused stress concentration.

### 3.4. Von Mises Stress on the Lower Endplate of T12 and the Upper Endplate of L2

Table 6 shows that the maximum von Mises stresses on the two endplates were almost the same as each other among the six types of constructs under flexion and extension loading. However, under lateral bending and axial rotation loading, the PAPS constructs displayed more significant stress on the two endplates than the other two types.

## 4. Discussion

As known to all, lack of adequate anterior column support is an important reason for rekyphosis and early implant failure after the traditional posterior pedicle screw fixation [10]. To increase the stiffness of the entire instrument, several technical modifications have been made in the posterior pedicle screw construct, including crosslink, hook [24], and intermediate screw [25] or vertebral augmentation at the fracture level [10]. In Liao et al.'s study [10], the crosslink could relieve the stress of the whole construct under a rotation load, which cannot be used in the minimally invasive procedure.

The intermediate screw has been verified to be an efficient reinforcer to the biomechanical stability of the whole construct. Baaj et al. [26] and Mahar et al. [27] compared 6-MAPS versus 4-MAPS/2-NIS construct using the cadaveric lumbar burst fracture model. Their results showed that the intermediate screw could decrease the ROM of the spine [26] and increase the stiffness of the construct [27]. Wang et al.'s in vitro test showed that the intermediate screw of the 6-MAPS construct could decrease more ROM of the burst fractured spine than the 6-PAPS construct [28]. The biomechanical superiority of the intermediate screw was also demonstrated in other FEA studies [10, 15, 17].

Li et al.'s FEA study compared the 6-MAPS versus 4-MAPS/2-NIS construct on the T11-L1 FE model and concluded that the maximum stress was located at the root of the upper pedicle screw under all loading conditions with 500 N compression and 6 Nm moments [29]. Liao et al. only applied 7.5 Nm moments to the 6-MAPS construct of the T11-L1 FE model and concluded that the maximum stresses were mainly on the screw's root under flexion and extension and the rod under lateral bending [10]. However, Basaran et al. argued that the greatest stress of the T11-L1 6-MAPS construct in the T9-L3 FE model was seen on the upper screw under flexion and axial rotation, but on the lower screw under lateral bending and axial rotation [17]. Wang et al.'s FEA study compared 6-MAPS with the 6-PAPS construct and concluded that the maximum von Mises stress was seen at the lower screw of the 6-PAPS construct under extension and the upper screw of the 6-MAPS construct under flexion [30].

Until now, there were limited FEA studies on the UPPS system and hardly any intermediate screw used in the UPPS short-segment construct either. To facilitate vertebral augmentation, we designed a new intermediate screw with a new screw configuration. Our FEA results showed that the biomechanical performance of the UPPS short-segment construct with the NIS was acceptable by comparing with 6-UPPS or 4-MAPS/2-NIS. Our results were not entirely the same as the previous FEA studies because of inconsistent conditions and parameters. We did not see the maximum stress on the rod like Wang et al. [30]. The possible reason might be the preloading vertical compression with 500 N in our study that was much greater than 150 N in theirs [30]. However, the positions of maximum stresses on six-screw constructs were comparable to their four-screw counterparts with the NIS, which also indirectly validated our established FE model.

Table 2 shows that the maximum von Mises stresses on the NIS were less than their counterparts in the six-screw constructs with the parallel configuration. The possible reason might be that the NIS was deviated from the plane on which the upper and lower pedicle screws were located. Thus, with different types of loading, the NIS accepted less stress than that in the parallel configuration, especially under flexion and extension. This difference became less significant in the PAPS groups because of its screw-tulip universal joint. However, under axial rotation, the maximum von Mises stress was extremely large on the NIS in the 4-PAPS/2-NIS construct. The reason might be that the NIS was rigidly connected with the rod rather than the mobile joints of the upper and lower PAPS. Under flexion, extension, and lateral bending, this connection difference between the NIS and upper/lower PAPS was not so significant; however, under the axial rotation moment, without the longitudinal rod to bear the loading, this difference displayed a significant effect on 4-PAPS/2-NIS with the maximum von Mises stress of 256.62 MPa on the NIS than 66.09 MPa on the intermediate screw of the 6-PAPS construct.

One potential advantage of our new construct is that the smooth middle one-third shank of the NIS allowed increased ROM of the screw within the injured vertebra, which could conveniently elevate the endplate of the injured vertebra and strengthen the fracture fixation simultaneously. Another advantage is the combination of vertebroplasty and the intermediate screw. The conventional short-segment construct does not allow additional vertebroplasty without interfering its standard procedure. The current solutions include a short-segment construct with vertebroplasty but without the intermediate screw [13] or partial vertebroplasty with a short intermediate screw [10, 31]. In the studies on the six-screw construct combined with bone grafting [14] or vertebroplasty [12], after the reduction maneuver, the intermediate screw needed to be removed and reinserted at the interval between the augmentation and fixation steps, which interrupted the normal operation process and extended the operative time. Korovessis et al. performed minimally invasive 6-MAPS short-segment fixation with kyphoplasty in A2/A3 lumbar fractures [31]. However, the short intermediate screws could only be inserted partly into the fractured vertebra after kyphoplasty. Neither of the two alternatives can give full play to both technologies.

Our study had several limitations. First, although there existed several potential advantages, including vertebroplasty and the elevation and augmentation of the depressed endplate with the help of the NIS, we did not prove it in the present study due to the study design and space limitation. The application in minimally invasive spinal fixation with vertebral augmentation should be conducted in further simulated and clinical studies. Second, the FE model was based on a healthy male. How our system performs in the vertebral osteoporotic compression fracture is still needed to be determined. Third, our FE model was simplified without considering all the influencing factors. For example, the endplates were considered a part of the cortical bone rather than an independent structure. In vitro experiments on cadaveric specimen should be performed to verify the results achieved in the present study.

## 5. Conclusions

The observations shown in this study verified that the developed hybrid uniplanar pedicle screw system exhibited comparable biomechanical performance than the current posterior short-segment constructs. Considering the biomechanical performance and superiority of vertebral augmentation and operation maneuvers, this new fixation system may provide researchers and clinical practitioners an important alternative for minimally invasive spinal fixation in patients with thoracolumbar vertebral compression fracture.

## Figures and Tables

**Table 1:** The dimensions of vertebrae and intervertebral disc (IVD).

Vertebra	a	b	c	d	e	IVD	f	g
T12	38.53	67.75	29.52	67.79	24.17	T12-L1	4.97	2.81
L1	40.88	69.25	30.56	68.58	25.35	L1-L2	6.86	3.72
L2	40.45	75.13	29.01	69.17	25.83			

**Table 2:** Material properties in the FEA.

Spinal site	Young's Modulus (MPa) [10, 15]	Poisson's ratio
Cortical bone/endplate	12000	0.3
Cancellous bone	100	0.2
Annulus	4.2	0.4
Nucleus pulposus	1	0.49
Posterior element	3500	0.25
Pedicle screws and rods	110000	0.3

**Table 3:** ROMs of intact T12-L1 in the present study and references.

Motion type	T12-L1		L1-L2	
	FEA	[21]	FEA	[22]
Flexion-extension	8.49	6.78	8.42	9.20
Left-right lateral bending	4.34	3.7	8.73	8.90
Axial rotation	1.68	1.19	3.60	3.90

**Table 4:** Range of motion (ROM) (°) of T12-L2 and maximum displacement (mm) of T12 after six types of the pedicle screw fixation under different types of loading.

Fixation types	ROM of T12-L2				Maximum displacement of T12			
	Flexion	Extension	RLB	RAR	Flexion	Extension	RLB	RAR
6-MAPS	3.89	1.86	4.34	2.35	2.31	0.71	1.93	1.96
6-UPPS	3.88	2.89	5.15	2.53	2.28	0.74	2.06	2.29
6-PAPS	3.94	3.64	6.06	3.5	2.32	0.89	2.31	3.55
4-MAPS/2-NIS	3.84	2.22	4.35	2.37	2.28	0.73	1.94	1.98
4-UPPS/2-NIS	3.96	2.79	5.27	2.52	2.33	0.78	2.11	2.28
4-PAPS/2-NIS	3.94	3.19	5.98	2.58	2.32	0.84	2.28	2.62

RLB: right lateral bending; RAR: right axial rotation; 6-MAPS: six monoaxial pedicle screw fixation; 6-PAPS: six polyaxial pedicle screw fixation; 6-UPPS: six uniplanar pedicle screws fixation; 4-MAPS/2-NIS: four monoaxial pedicle screw fixation with two new intermediate screws; 4-PAPS/2-NIS: four polyaxial pedicle screw fixation with two new intermediate screws; 4-UPPS/2-NIS: four uniplanar pedicle screw fixation with two new intermediate screws.

**Table 5:** Maximum von Mises stress (MPa) on the screw and rod of the construct under different types of loading.

Screw position/rod	Flexion						Extension					
	6-MAPS	6-UPPS	6-PAPS	4-MAPS/ 2-NIS	4-UPPS/ 2-NIS	4-PAPS/ 2-NIS	6-MAPS	6-UPPS	6-PAPS	4-MAPS/ 2-NIS	4-UPPS/ 2-NIS	4-PAPS/ 2-NIS
Upper	*188.03*	*138.04*	96.627	*202.93*	*145.23*	96.439	107.68	66.724	126.17	127.42	75.025	124.17
Intermediate	96.809	78.74	83.935	55.664	58.019	61.909	75.384	38.466	49.169	18.843	20.919	48.863
Lower	166.75	108.1	*100.74*	152.28	107.65	*118.27*	*127.32*	*187.86*	*206.74*	*135.73*	*192.08*	*195.52*
Rod	109.66	71.177	58.335	119.11	95.562	86.133	116.23	76.532	55.825	113.85	81.163	68.775
Screw position/rod	Right lateral bending						Right axial rotation					
	6-MAPS	6-UPPS	6-PAPS	4-MAPS/ 2-NIS	4-UPPS/ 2-NIS	4-PAPS/ 2-NIS	6-MAPS	6-UPPS	6-PAPS	4-MAPS/ 2-NIS	4-UPPS/ 2-NIS	4-PAPS/ 2-NIS
Upper	*224.7*	148.89	218.58	*244.2*	161.27	144.66	*361.95*	*301.43*	28.544	*367.55*	*296.67*	56.743
Intermediate	103.91	62.373	58.86	41.66	42.455	61.692	310.21	263.81	66.087	119.46	170.4	*256.62*
Lower	182.93	*205.77*	*272.05*	166.83	*211.47*	*272.7*	289.38	190.43	*79.318*	277.75	187.06	105.01
Rod	180.69	126.13	74.584	179.06	136.06	88.923	132.01	111.46	62.264	147.51	146.31	225.1

6-MAPS: six monoaxial pedicle screw fixation; 6-PAPS: six polyaxial pedicle screw fixation; 6-UPPS: six uniplanar pedicle screw fixation; 4-MAPS/2-NIS: four monoaxial pedicle screw fixation with two new intermediate screws; 4-PAPS/2-NIS: four polyaxial pedicle screw fixation with two new intermediate screws; 4-UPPS/2-NIS: four uniplanar pedicle screw fixation with two new intermediate screws. The maximum von Mises stress of the entire construct under loading was shown as *italics*.

**Table 6:** Maximum von Mises stress (MPa) on the lower endplate of T12 and the upper endplate of L2 under different types of loading.

Fixation types	Stress on the lower endplate of T12				Stress on the upper endplate of L2			
	Flexion	Extension	RLB	RAR	Flexion	Extension	RLB	RAR
6-MAPS	11.86	11.45	15.66	8.74	10.82	15.20	14.85	9.76
6-UPPS	11.67	11.36	16.26	9.43	10.51	15.50	15.78	10.29
6-PAPS	12.10	11.63	18.55	15.84	10.51	16.11	16.97	14.92
4-MAPS/2-NIS	11.62	11.34	15.38	8.51	10.97	15.27	14.91	9.86
4-UPPS/2-NIS	11.89	11.60	16.44	9.15	10.66	15.95	16.24	10.71
4-PAPS/2-NIS	12.06	11.67	18.16	12.89	11.65	15.65	17.25	12.33

RLB: right lateral bending; RAR: right axial rotation; 6-MAPS: six monoaxial pedicle screw fixation; 6-PAPS: six polyaxial pedicle screw fixation; 6-UPPS: six-uniplanar pedicle screw fixation; 4-MAPS/2-NIS: four monoaxial pedicle screw fixation with two new intermediate screws; 4-PAPS/2-NIS: four polyaxial pedicle screw fixation with two new intermediate screws; 4-UPPS/2-NIS: four uniplanar pedicle screw fixation with two new intermediate screws.

## Data Availability

The data of the FE model validation, ROM of T12-L2 and maximum displacement of T12, and von Mises stress on the pedicle screw system and endplate were used to support the findings of this study that are included within the article.

## Supplementary Materials

Supplementary Materials Six-monoaxial pedicle screw fixation, six uniplanar pedicle screw fixation, six polyaxial pedicle screw fixation, four monoaxial pedicle screw fixation with two new intermediate screws, four uniplanar pedicle screw fixation with two new intermediate screws, and four polyaxial pedicle screw fixation with two new intermediate screws.
