# Supplementary material for: A Hybrid Uniplanar Pedicle Screw System with a New Intermediate Screw for Minimally Invasive Spinal Fixation: A Finite Element Analysis
Source: Biomed Res Int. 2020 Nov 18;2020:5497030. doi: 10.1155/2020/5497030 (PMC7691004; doi:10.1155/2020/5497030)

## Six monoaxial pedicle screws fixation


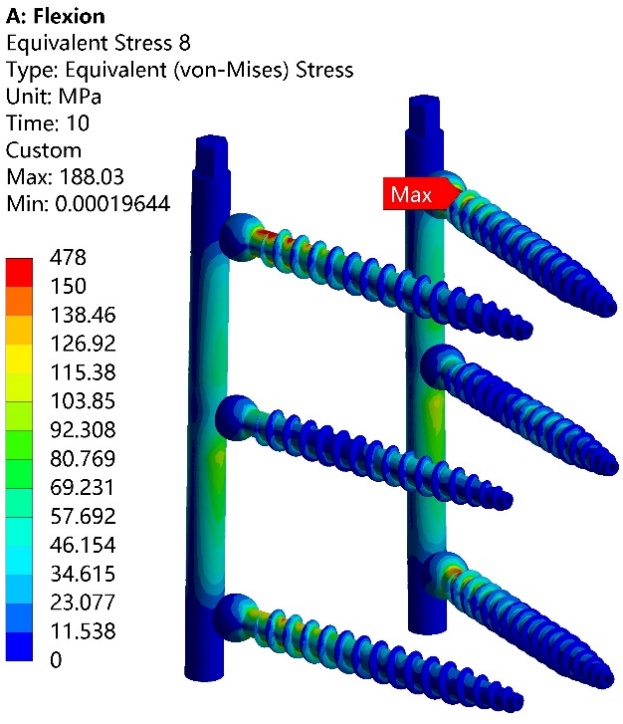

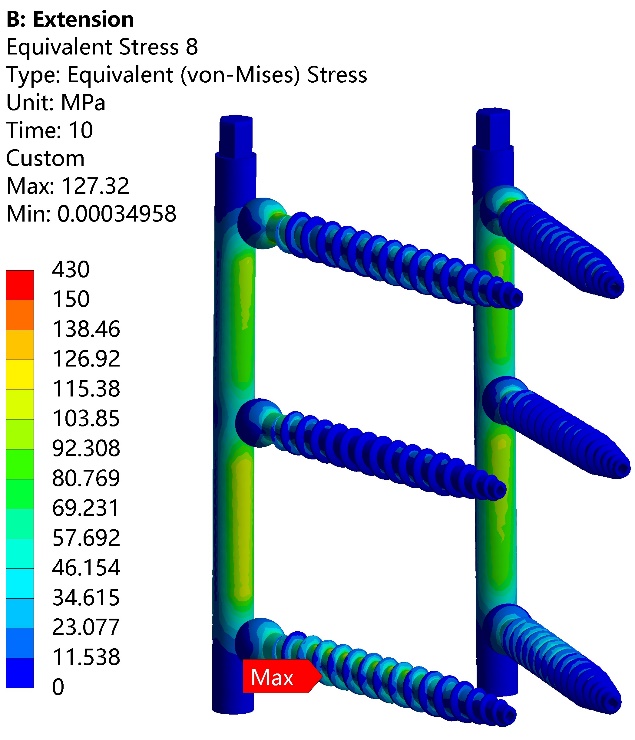


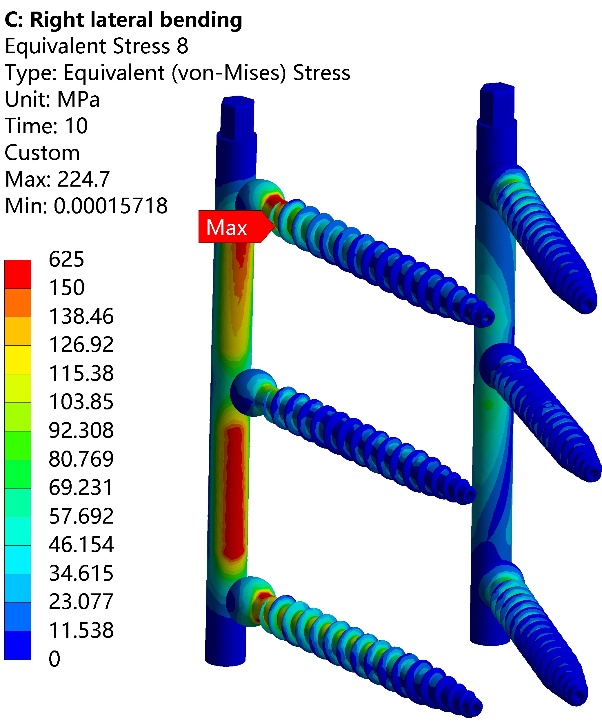

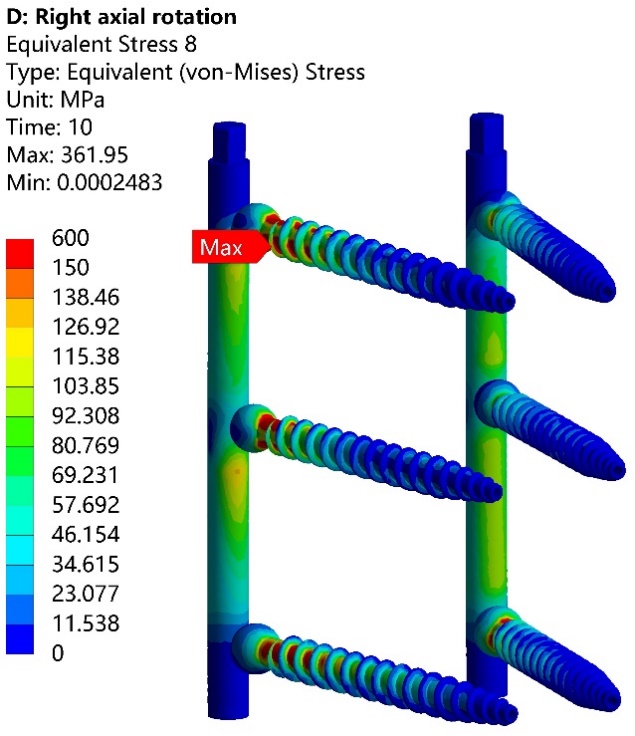


## Six uniplanar pedicle screws fixation


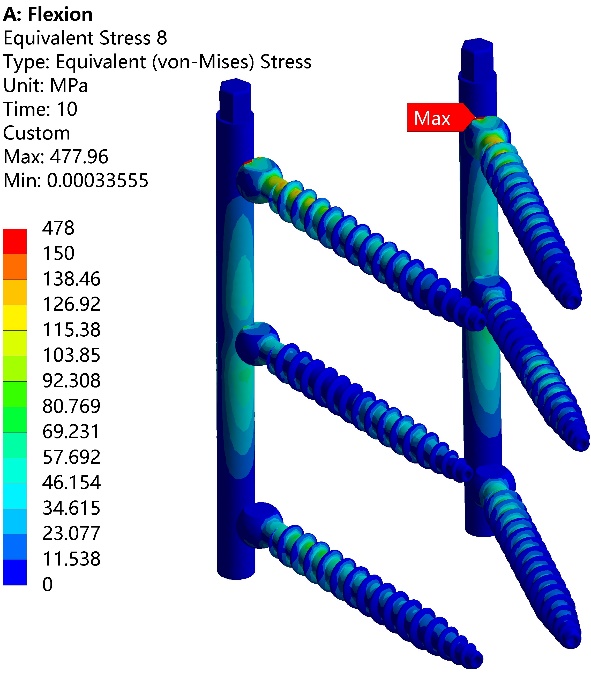

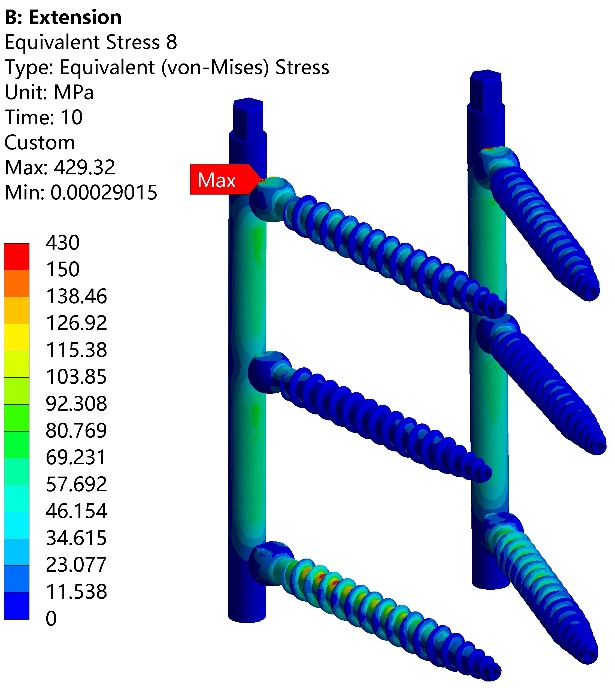


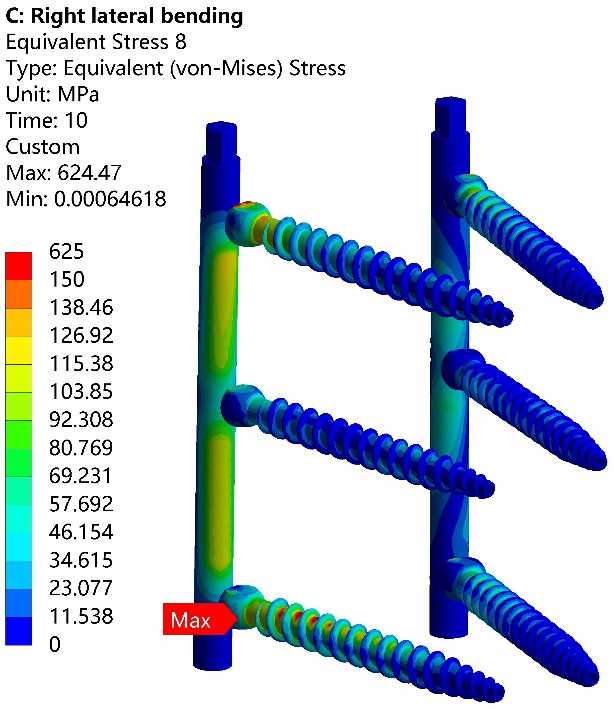

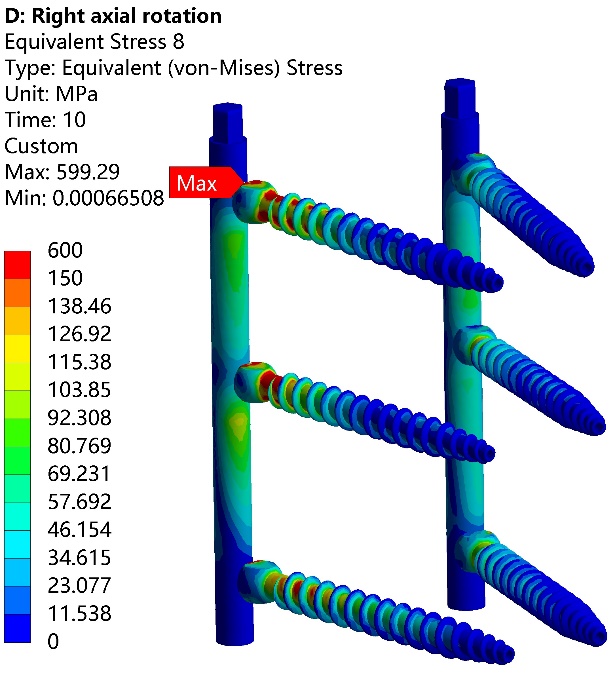


## Six polyaxial pedicle screws fixation


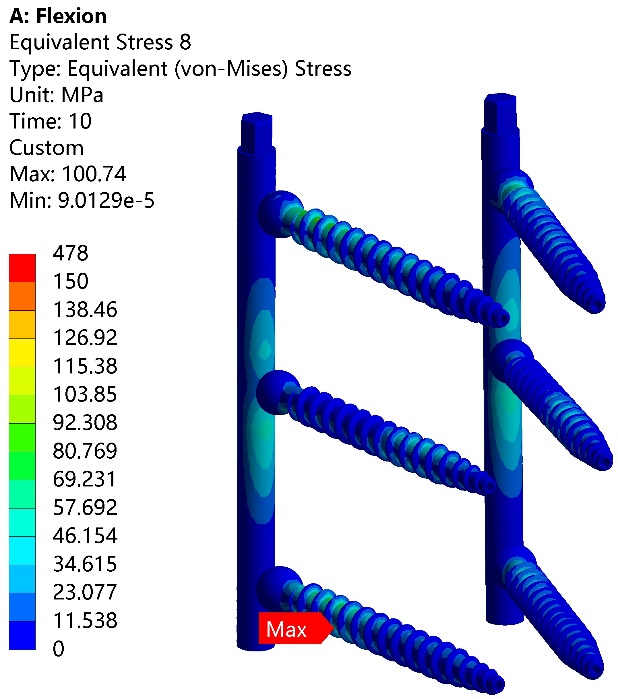

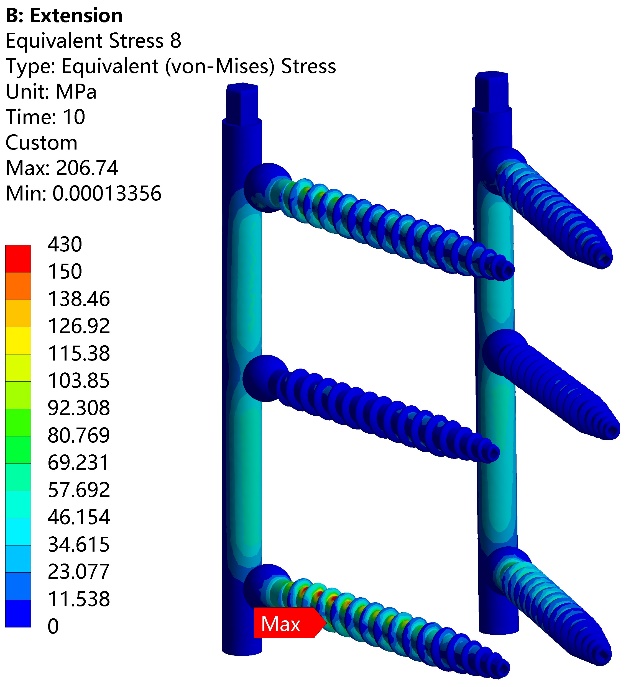


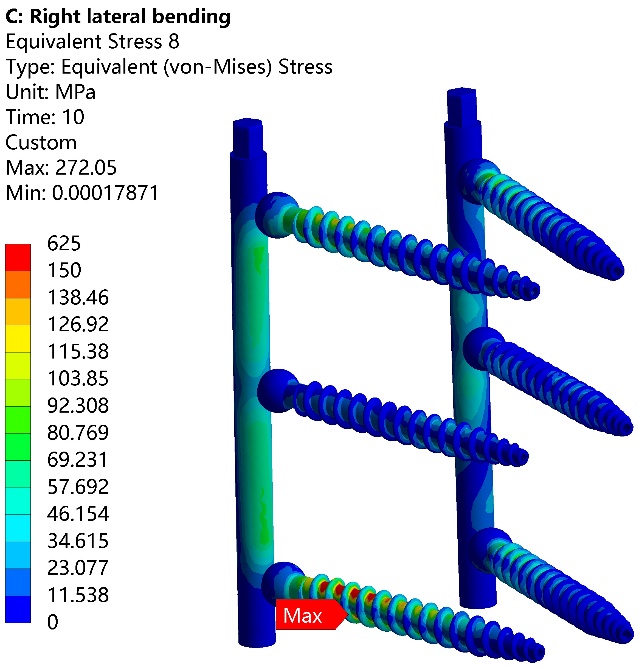

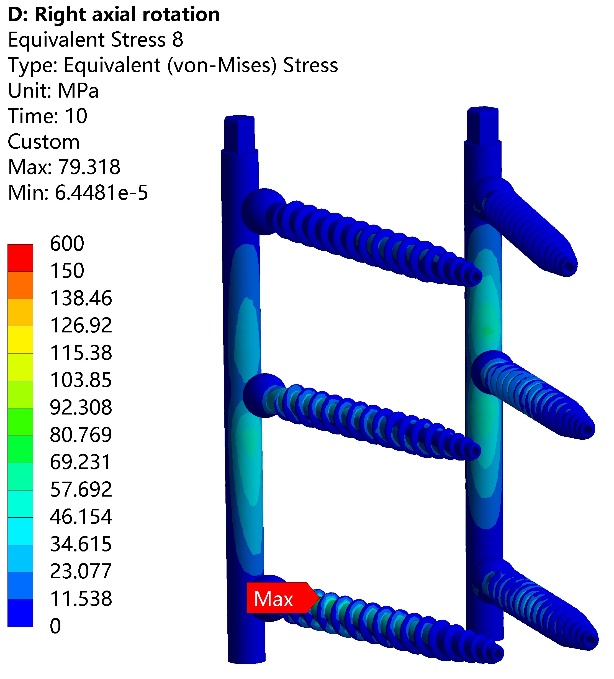


## Four monoaxial pedicle screws fixation with two new intermediate screws


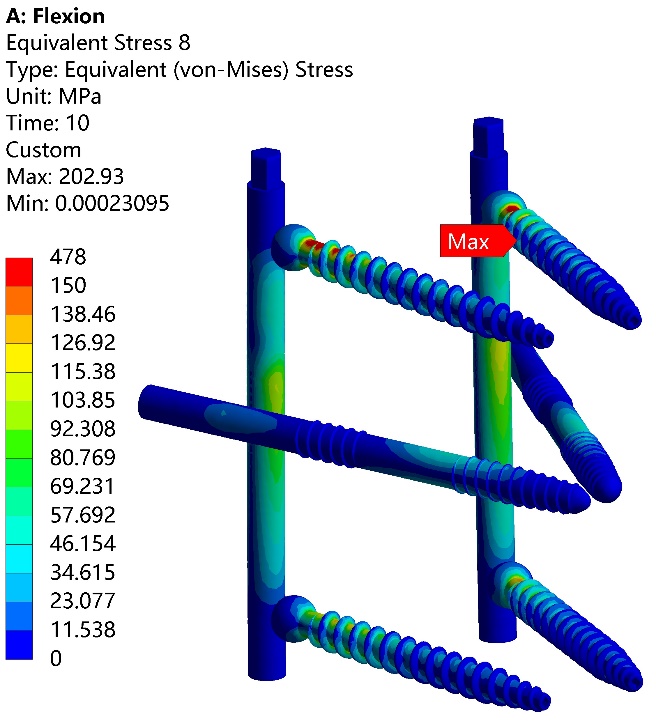

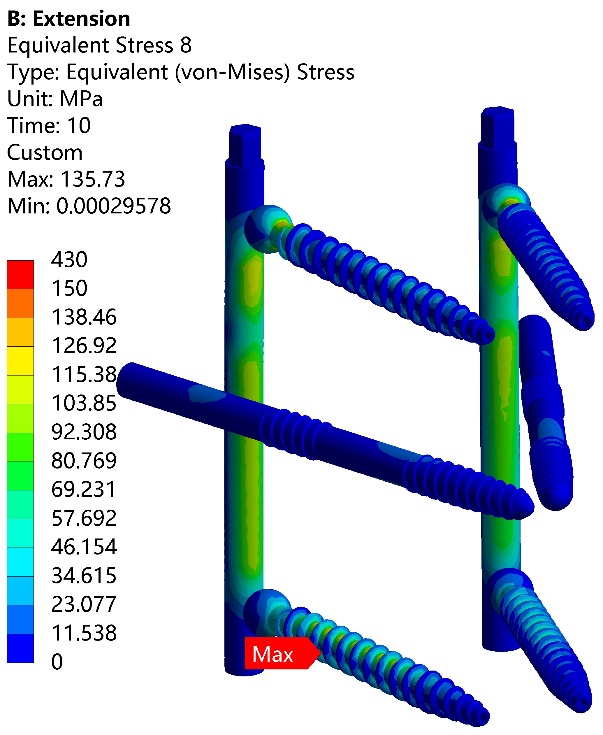


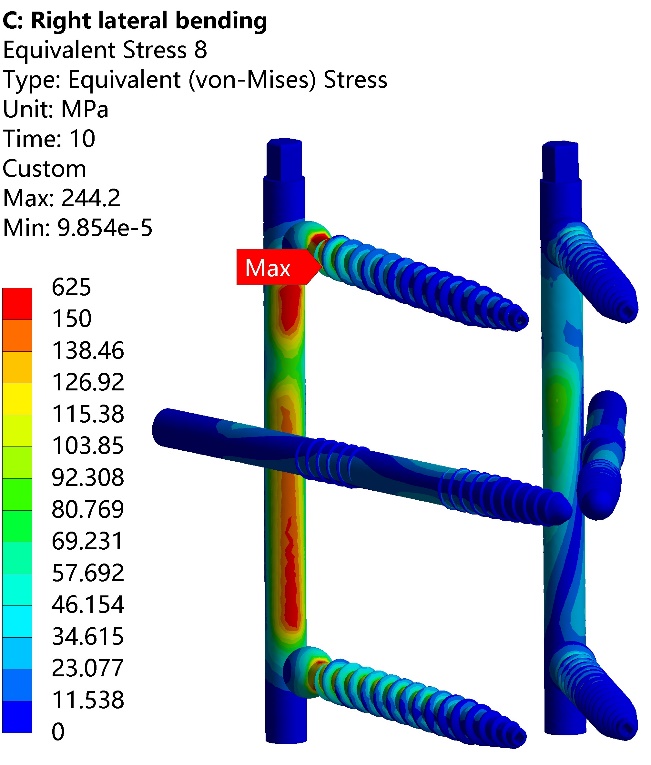

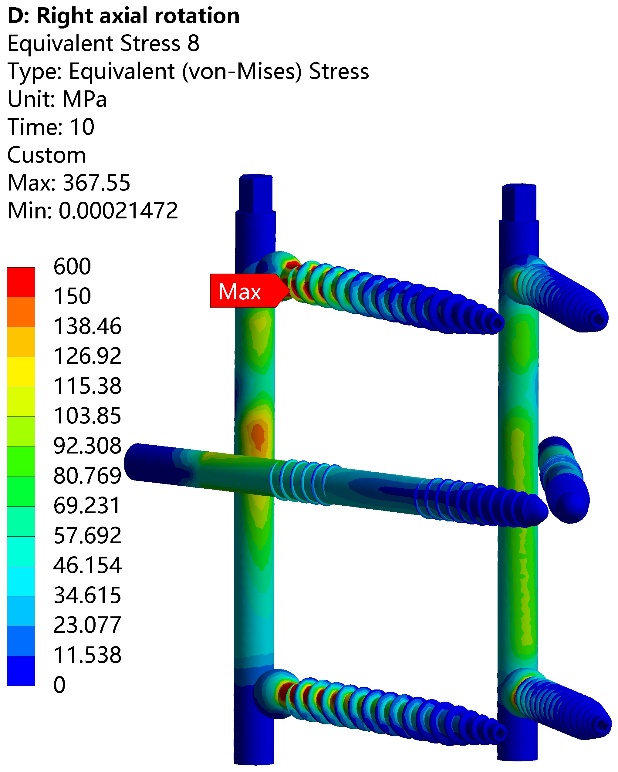


## Four uniplanar pedicle screws fixation with two new intermediate screws


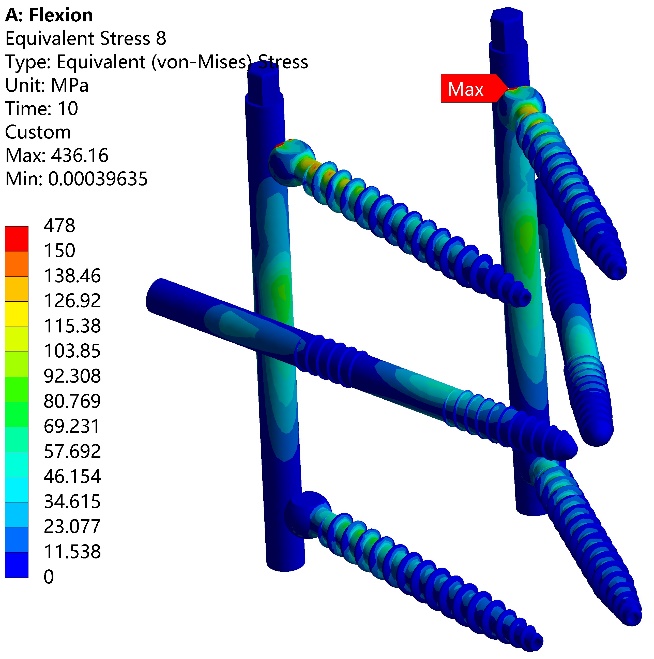

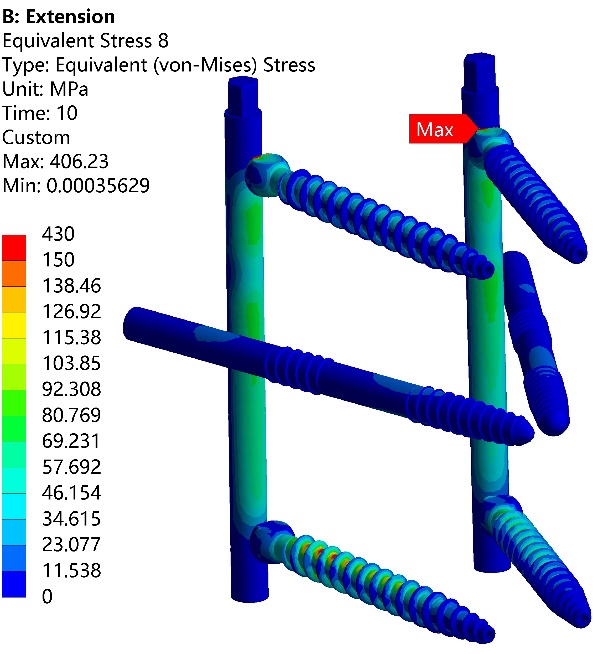


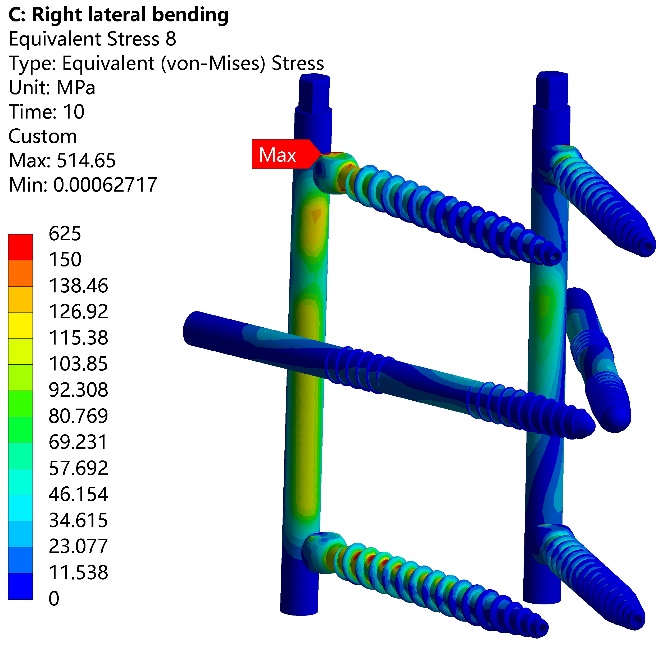

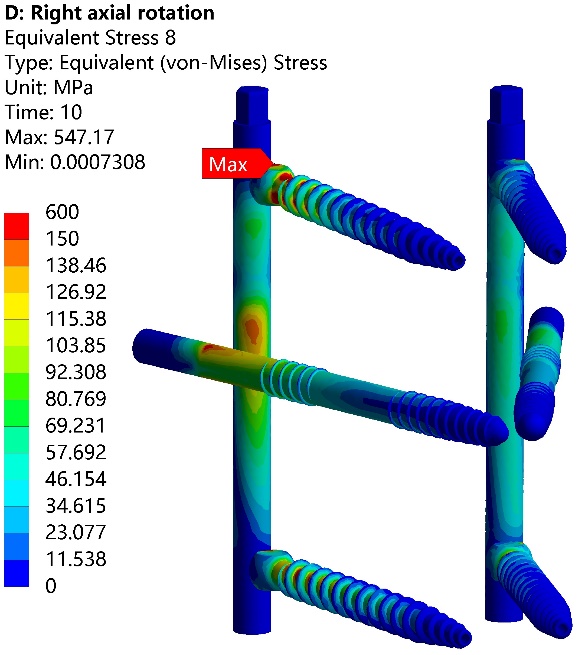


## Four polyaxial pedicle screws fixation with two new intermediate screws


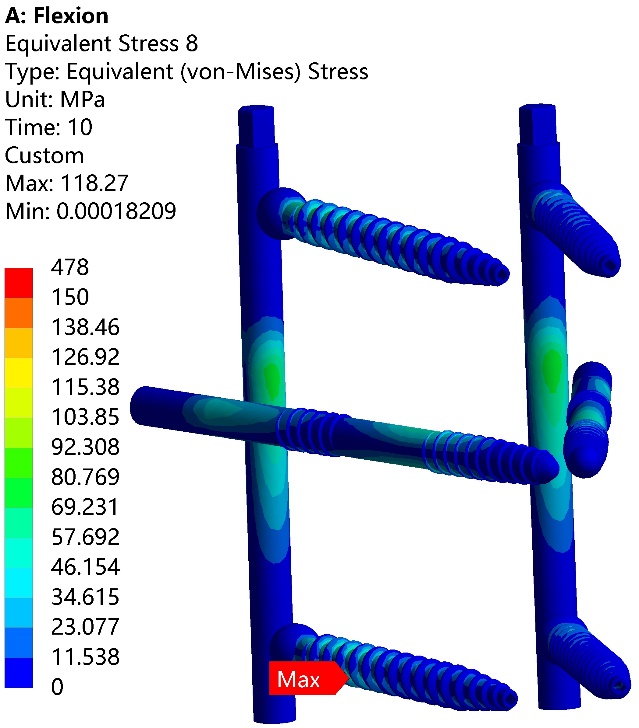

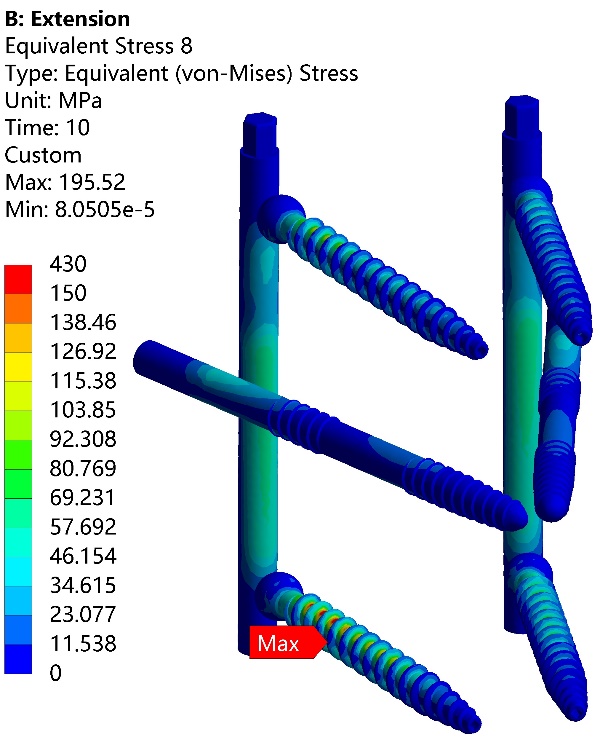


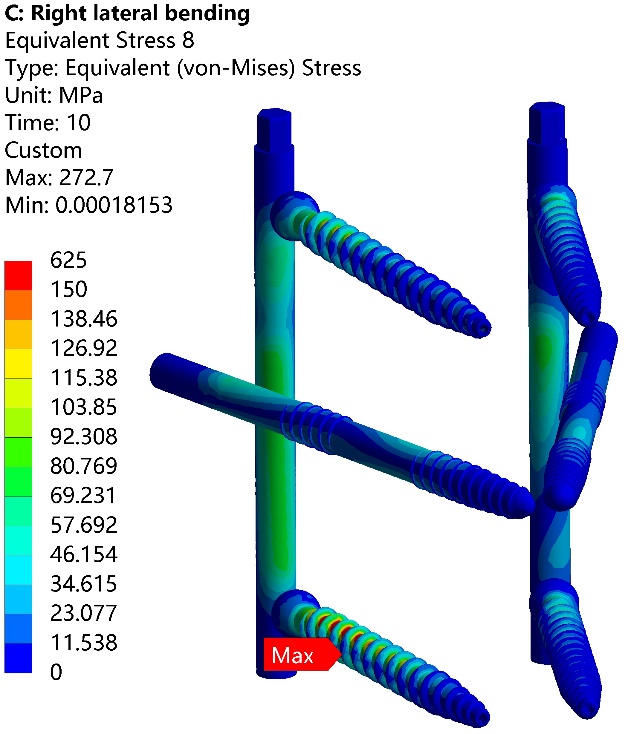

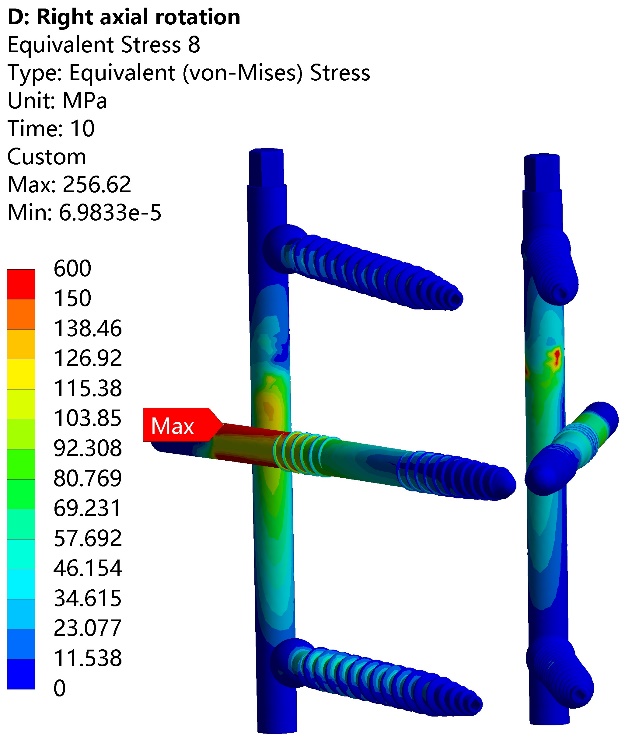

Supplement: Supplementary Materials — Six-monoaxial pedicle screw fixation, six uniplanar pedicle screw fixation, six polyaxial pedicle screw fixation, four monoaxial pedicle screw fixation with two new intermediate screws, four uniplanar pedicle screw fixation with two new intermediate screws, and four polyaxial pedicle screw fixation with two new intermediate screws. [file 5497030.f1.docx]
